# Early Osteogenic Differentiation Stimulation of Dental Pulp Stem Cells by Calcitriol and Curcumin

**DOI:** 10.1155/2021/9980137

**Published:** 2021-05-23

**Authors:** Mohammad Samiei, Atefeh Abedi, Simin Sharifi, Solmaz Maleki Dizaj

**Affiliations:** ^1^Stem Cell Research Center, Tabriz University of Medical Sciences, Tabriz, Iran; ^2^Department of Endodontics, Faculty of Dentistry, Tabriz University of Medical Sciences, Tabriz, Iran; ^3^Dental and Periodontal Research Center, Tabriz University of Medical Sciences, Tabriz, Iran

## Abstract

Curcumin, as a natural phenolic substance, is extracted from the rhizome of Curcuma longa (turmeric), which is effective in bone healthfulness. Calcitriol is an effective hormone in regulating bone remodeling and mineral homeostasis and immune response. Mesenchymal stem cells (MSCs) are found in most dental tissues and resemble bone marrow-derived MSCs. In this work, we investigated the effect of combination and individual treatment of curcumin and calcitriol on early osteogenic differentiation of dental pulp stem cells (DPSCs). Early osteogenic differentiation was evaluated and confirmed by the gene expression level of *ALP* and its activity. Curcumin individually and in combination with calcitriol increased ALP activity and osteoblast-specific mRNA expression of *ALP* when DPSCs were cultured in an osteogenic medium. Calcitriol alone increased the enzyme more than in combination with curcumin. These findings demonstrate that curcumin can induce early osteogenic differentiation of DPSCs like calcitriol as a potent stimulant of osteogenesis.

## 1. Introduction

Herbal remedies provide the basis for the production of modern medicines [1–3]. Curcumin, a natural polyphenolic hydrophobic product, is extracted from the rhizomes of Curcuma longa. It has various medicinal properties including antioxidant and anti-inflammatory, antimicrobial, antifungal, antiviral and antiangiogenic, anticancer, and antiatherosclerotic effects [4–11].

The advantageous properties of curcumin on diseases such as obesity, osteolysis, osteoporosis, and osteosarcoma have been reported in that differentiation of stem cells has an important role [12]. Numerous investigations have shown that curcumin is useful for increasing osseous mineral density and enhancement of bone microarchitecture, decreases bone destruction due to ovariectomy, and inhibits osteoporosis and osteoarthritis [13–18]. These results indicate that curcumin may enforce bone remodeling by inhibiting or inducing osteocyte differentiation [6, 19].

MSCs are one of the main types of cells used in regeneration treatments, with bone marrow or adipose tissue being the main site of extraction. The multipotent stem cells in interaction with various factors such as vitamin D3 and bone morphogenic proteins (BMPs) and bone growth proteins form a broad set of tissues like bone and fat and cartilage to maintain the body composition balance [20].

Another type of MSCs that are isolated from dental pulp tissue is the DPSC. These pluripotent cells can be differentiated into different cell types such as osteoblasts, odontoblast, fibroblasts, chondroblasts, neuroblasts, adipocytes, and myoblasts [21–23]. DPSCs are an alternative source of MSCs and can be used in regenerative and tissue engineering processes to treat a variety of bony defects due to congenital malformations and tumors, trauma, and age-related osteoporosis [24, 25].

Oral-derived stem cells related with particular biomaterials are utilized in numerous tissue engineering processes currently [26–28].

In response to mechanical disruption, degradation, and erosion, it has been reported that DPSCs can differentiate into odontoblast-like cells and form dentin.

It has been shown that DPSCs can differentiate into odontoblast-like cells for dentin formation in response to disruption, degradation, and mechanical erosion. In fact, various studies have shown that mechanical stresses can affect the DPSC behavior [29, 30]. The differentiation and formation of odontoblast-like cells derived from DPSCs depend on environmental signal transduction comprising both physical and chemical stimulants [29, 31].

Vitamin D, as a fat-soluble secosteroid, is important for the improvement of the absorption of calcium, phosphate, magnesium, zinc, and iron. A group of vitamin D3 metabolites regulates osseous homeostasis and remodeling. The most active form of vitamin D3 is calcitriol, which has a critical role in the physiology of the body including promoting normal bone growth and improving neuromuscular and immune responses. The most important effect of calcitriol is maintaining the balance of calcium-phosphorus that affects bone health and also is crucial for calcification [32–34].

Calcitriol induces osteoblastic differentiation of human MSCs. This process can be assessed by increased ALP activity or *osteocalcin* (OCN) gene expression levels [35].

Considering the very important effect of calcitriol in ossification and stimulation of stem cells towards osteogenesis and the effect of curcumin as a useful and widely available plant derivative and its effect on osteogenic stimulation on mesenchymal stem cells, in this study, we decided to evaluate the effect of curcumin, calcitriol, and combination of them on early osteogenic differentiation of DPSCs.

## 2. Material and Methods

### 2.1. Material Preparation

For the preparation of calcitriol (1,25-dihydroxyvitamin D3) and curcumin solutions, powder of calcitriol (Rocaltrol™, Roche, Mannheim, Germany) was dissolved in ethanol (1 *μ*g/*μ*l) and powder of curcumin (Sigma-Aldrich, Steinheim, Germany) was dissolved in DMSO (10 *μ*g/*μ*l).

### 2.2. Cell Culture

Human DPSCs were achieved from Shaid Beheshti University of Medical Sciences in Iran, which was extracted from impacted wisdom teeth. The cells were cultivated in DMEM which was supplemented with 10% fetal bovine serum (FBS) and 1% streptomycin/penicillin and incubated with 5% CO_2_ at 37°C.

### 2.3. Cell Viability and Proliferation Assay

In this study, for evaluation of curcumin and calcitriol effect on viability and proliferation of DPSCs was used from MTT assay. Cells were cultured in 96-well plates (5000 cells/well) and treated with curcumin, which was dissolved in DMSO, at concentrations of 0.5, 1, 2.5, 5, 10, and 15 *μ*M, at times of 1, 2, 3, and 7 days. Experiments were performed at the same time for calcitriol at concentrations of 1, 2.5, 5, 10, 25, 50, and 100 *μ*M. Evaluation of viability for the combination of curcumin (0.5, 1, 2.5, and 5 *μ*M) with calcitriol (10 nM) were done after 7 days incubation. After the treatment time, washing was performed and incubated with 200 *μ*l of MTT solution (0.5 mg/ml) for 4 hours at 37° C and away from light. After this time, the above solution was extracted and 200 *μ*l of DMSO was added to each well and placed on a shaker for 15 minutes. Then, their absorbance at 570 nm was read by plate reader and the percentage of living cells was evaluated by comparing the control (cells grown in the absence of curcumin and calcitriol). All tests were performed in three replications. For the MTT experiment on the combination of curcumin (0.5, 1, 2.5, and 5 *μ*M) and calcitriol (10 nM), the appropriate concentrations of the two (concentrations that had no toxic effect but had a proliferative effect) were selected.

### 2.4. Osteogenic Differentiation Induction of Human DPSCs

Human DPSCs were exposed to osteogenic differentiation media: *α*-MEM medium supplemented with 100 IU/ml penicillin, 100 *μ*g/ml streptomycin, 10 nM dexamethasone and 0.2 mM sodium L-ascorbyl-2-phosphate, 10 mM *β*-glycerol phosphate, and 10% FBS (Gibco, Grand Island, NY). The differentiation medium was renewed every three days.

Curcumin (0.5 *μ*M) and calcitriol (10 nM) individually and in combination with each other were added to cells in the differentiation medium. The gene expression level and activity of ALP were determined on day 7.

### 2.5. RT Real-Time PCR

We used the RiboEx reagent (GeneAll, South Korea) to extract the total RNA of cells at the specified intervals. The cDNAs were obtained from the extracted RNAs by reverse transcription (Solis Biodyne, Tartu, Estonia) according to the manufacturer's protocols.

In order to carry out the quantitative real-time PCR, we used a SYBR Green dye-based detection technique (Solis BioDyne, Estonia), triplicated samples, and serial dilutions of control cDNA to create standard curves of each gene expression.

The particular primers were utilized containing: *ALP*, forward: 5′-GACCCTTGACCCCCACAAT-3′, reverse: 5′-GCTCGTACTGCATGTCCCCT-3′ and *GAPDH*, forward: 5′-AGCCACATCGCTCAGACAC-3′, reverse: 5′-GCCCAATACGACCAAATCC-3′.

The normalization of results was used from the expression of *GAPDH*.

### 2.6. ALP Activity Assay

After 7 days of treatment, the cells grown under osteogenic media were extracted and resuspended in 250 *μ*l of culture supernatants; then, an ultrasound breaker was used to cell break. Followed by centrifugation, supernatant of cells was used for quantification of the ALP activities via a special detection kit (Nanjing Jiancheng Biotechnology Institute, Nanjing) and a spectrophotometer (Bio-Rad, Hercules, CA) at a wavelength of 520 nm. The relative ALP activity was normalized to the protein concentration.

## 3. Results

**Figure 1:**
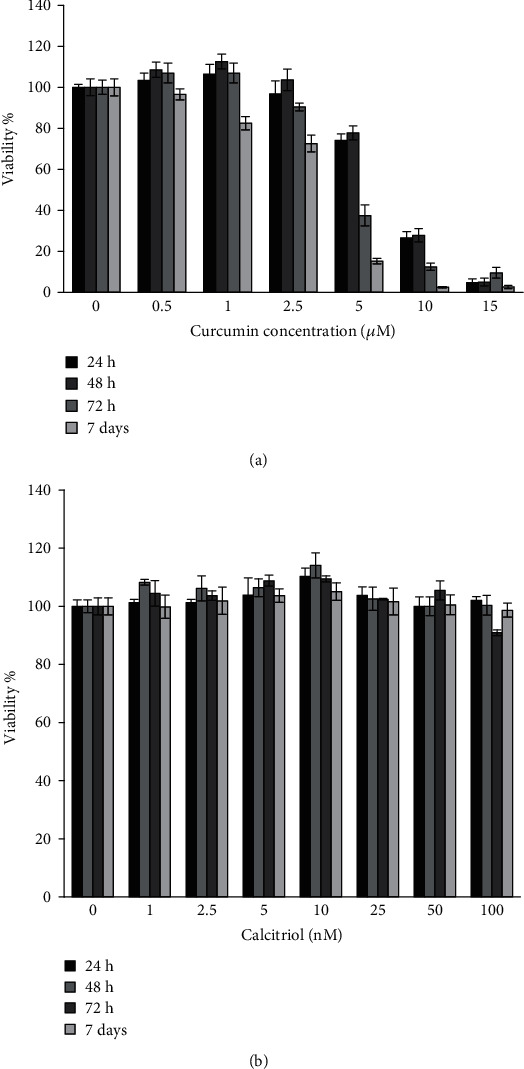
(a) Viability of DPSCs after treatment with curcumin in 24, 48, and 72 h and 7 days. (b) Viability of DPSCs after treatment with calcitriol in 24, 48, and 72 h and 7 days.

We analyzed the DPSC viability after treatment with curcumin by MTT proliferation assay. (Figure 1(a)). After 24, 48, and 72 hours of incubation, cell viability was increased significantly in the curcumin low concentrations (0.5 and 1 *μ*M) (*P* < 0.05), and there was no significant difference between them. However, cell viability was decreased significantly in high concentration (5, 10, and 15 *μ*M) (*P* < 0.05).

Also, we analyzed the DPSC viability after treatment with calcitriol (Figure 1(b)). After 24, 48, and 72 hours and 7 days of incubation with calcitriol, cell viability was increased significantly in the concentration of 10 nM (*P* < 0.05). However, cell viability was not decreased significantly in all concentrations (*P* < 0.05).

**Figure 2:**
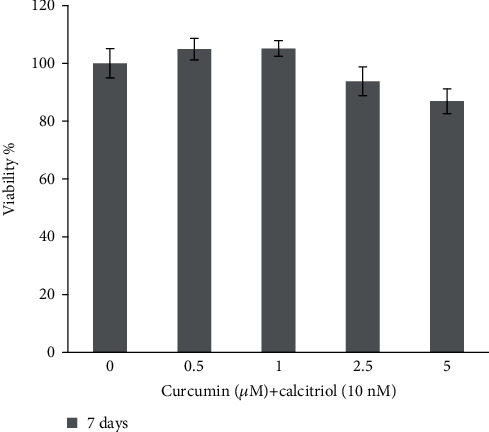
Viability of curcumin and calcitriol combination in 7 days.

The results of the MTT assay to evaluate the cytotoxicity of curcumin and calcitriol for 7 days showed that the combination of these two substances in concentrations of 0.5 and 1 *μ*M of curcumin and 10 nM calcitriol did not have a toxic effect on pulp stem cells but caused increased growth of these cells (Figure 2). In other words, the combination of 10 nM calcitriol and 0.5 and 1 *μ*M curcumin had a proliferative effect on DPSCs (*P* < 0.05). Also, during 7 days of exposure of DPSCs to 10 nM calcitriol and 5 *μ*M curcumin, it had a toxic effect on DPSCs.

**Figure 3:**
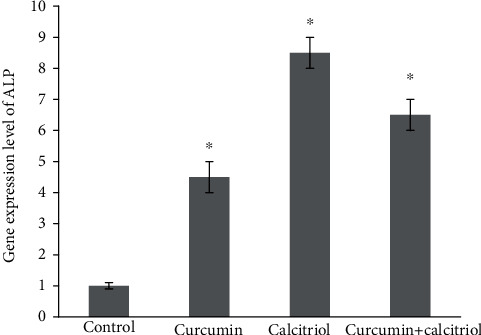
*ALP* gene expression on DPSCs treated with the combination of curcumin and calcitriol in 7 days.

The results of the *ALP* gene expression on DPSCs exposed to 0.5 *μ*M curcumin, 10 nM calcitriol, and combination of them for 7 days displayed a significant increase in the *ALP* gene expression compared to the control group (*P* < 0.05) (Figure 3).

**Figure 4:**
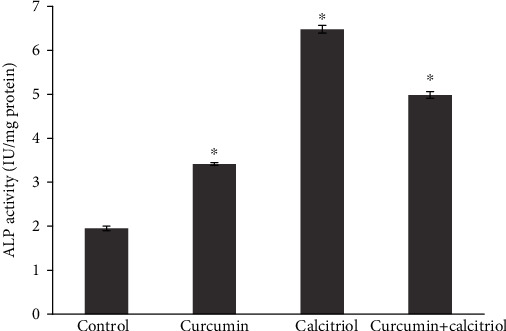
ALP enzyme activity on DPSCs treated with curcumin, calcitriol, and combination of them in 7 days.

The results of the ALP activity test on DPSCs treated with 0.5 *μ*M curcumin, 10 nM calcitriol, and combination of them for 7 days displayed a significant increase in the ALP activity compared to the control group (*P* < 0.05) (Figure 4).

## 4. Discussion

DPSCs are utilized in tissue engineering and bone regeneration, which has been successful in osteoporosis. While studies have shown that DPSCs enhance bone regeneration *in vitro* and *in vivo*, however, large-volume bone formation by DPSC-based therapy is still under investigation [36]. It is necessary to find a way to reset the DPSC capability for extensive clinical applications. There is evidence that curcumin and calcitriol may play a role in stem cell mineralization, so this study offers a new strategy to increase differentiation and induce DPSC mineralization [37–39].

The results of the present study exhibited that calcitriol did not have a toxic effect on DPSCs at all concentrations and test times (except at a concentration of 100 nM in 72 hours). These results also indicate that calcitriol has caused the growth and proliferation of DPSCs. Vitamin D is an important regulator of bone metabolism and development, participates in calcium homeostasis, and significantly increases the formation of bone in the absorbable area of the alveolar bone. Vitamin D can enhance the repair of supporting tooth tissue after orthodontic treatment. In addition, the active form of vitamin D has been shown to play a very important role in bone tissue because it can both induce bone formation and reabsorption and regulate bone turn over in osteoblasts and osteoclasts [40]. The attendance of vitamin D receptors directly affects osteoblasts, but these effects vary depending on the dose and time of treatment and the origin of the osteoblasts. Vitamin D primarily stimulates mineralization and differentiation of human osteoblasts [41].

In the study of Ji et al., the activity of inducing osteogenic differentiation in stem cells of human periodontal ligament origin by calcitriol was investigated. The results showed that the reduction of cell proliferation compared to the control group did not occur at different concentrations (0.01, 1 and 10 nm) for 24, 48, and 72 hours [42]. Wang et al. studied the effect of calcitriol (0.01 and 0.02 nm) on osteoblasts for 7 to 14 days and reported a significant increase in osteoblast survival [43].

Contrary to another study using MC3T3-E1 cells such as vitamin D-treated osteoblasts showed an increase in proliferation rate. Nevertheless, an increase in the expression of markers related to cell differentiation was also detected [44]. The recent finding is consistent with the results obtained in our study because, in the present study, the addition of calcitriol did not inhibit cell growth and proliferation. Hence, the actions of vitamin D are directly dependent on the doses and cause osteogenic differentiation of stem cells. Nevertheless, depending on the environmental conditions, the concentration and type of cell may also cause a significant change in cell proliferation.

In this study, the cytotoxicity of curcumin by MTT test showed that curcumin at low concentrations (0.5 and 1) for 24, 48, and 72 hours has no toxic effect on the DPSCs, but at the concentrations of 5, 10, and 15 *μ*M, curcumin has a toxic effect on the pulp stem cells of the tooth. Curcumin may exert cytotoxic effects by modulating oxidative stress parameters such as oxygen-reactive species, and the antioxidant genes expression in a time- and dose-dependent manner. The findings of the present study also showed that the combination of 0.5 *μ*M curcumin and 10 nM calcitriol for 7 days did not have a toxic effect on pulp stem cells but increased the growth of these cells. However, at the same time, exposure of DPSCs and increasing the concentration of curcumin to 5 *μ*M showed a toxic effect. The appropriate doses and times obtained from the results were used to measure the *ALP* gene expression and activity. Although there is recently no single specialized marker for DPSC differentiation, the expression of several markers is intended to study the differentiation process. Evaluation differentiation is performed by evaluating the expression of several specific genes. DPSCs have been reported to express typical osteoblastic markers including ALP, osteopontin (OPN), and collagen type I (Col I) and can produce mineral matrix in osteoblast-like cells [45]. However, ALP is a more prevalent protein, which is involved in osteogenesis and extracellular matrix mineralization.

Numerous studies have shown that this substance is a marker for early detection of osteogenic differentiation, in which the expression of gene and protein of ALP are greatly increased in differentiation into the bone and are therefore significantly associated with mineralization activity. After two days of stimulation, ALP mRNA levels increase in parallel with the process of osteogenic differentiation. In addition, ALP is an ectoenzyme that is involved in the release of mineral phosphate during mineral differentiation and, therefore, is an important marker for bone turnover [46]. Therefore, in this study, the gene and activity of ALP enzyme in DPSCs treated with curcumin and calcitriol were investigated. The results of the present study showed that the *ALP* gene expression and activity in DPSCs treated with 0.5 *μ*M curcumin, 10 nM cholesterol, and the combination of them in 7 days displayed a significant increase compared to the control group (*P* < 0.05).

Studies have shown that curcumin increases the osteogenic differentiation of stem cells. These results are demonstrated by testing for the gene expression or ALP activity level. The study by Son et al., which examined the cytotoxicity of curcumin and the expression of osteogenic markers in C3H10T1/2 mesenchymal stem cells, found that curcumin increased the expression of the *ALP* and *OCN* genes, which subsequently differentiated C3H10T1/2 cells. Curcumin also caused mild stress on the endoplasmic reticulum, such as the function of BMP2 in osteoblast cells [47]. Although the differentiation of curcumin-induced osteoblasts has been studied, there is no report of its mechanism at the molecular level.

Calcitriol alone increased the enzyme more than in combination with curcumin. These findings demonstrate that curcumin can induce early osteogenic differentiation of DPSCs like calcitriol as a potent stimulant of osteogenesis.

The present study has some limitations which we did not evaluate the expression of other osteogenic or odontogenic markers, such as DSPP, ON, RUNX2, and OCN, because evaluating them needs a long treat time (21 days), but we were able to treat the cells for only one week. Another limitation includes the lack of in vivo experiments; therefore, additional studies via animal models are essential in the future.

## 5. Conclusion

The increase in the ALP activity and the upregulation of the expression of ALP shown by curcumin and calcitriol indicate their potential to favor the differentiation of DPSCs. However, the expression of other osteogenic or odontogenic markers was not evaluated so further research is needed to confirm their influence on osteo/odontogenic differentiation.

Due to the positive effects of curcumin and calcitriol on the ALP activity and gene expression in DPSCs, these materials can be an option in bone and dental tissues engineering as an ingredient of scaffolds. Furthermore, curcumin is an active herbal ingredient that is inexpensive and available and can be an alternative to calcitriol to stimulate skeletal bone. It can also be used in the design of materials used to increase ossification, such as guided bone regeneration (GBR).

The take-home messages of the present study include the following: (1) curcumin individually and in combination with calcitriol increased ALP activity and osteoblast-specific mRNA expression of *ALP* when DPSCs were cultured in an osteogenic medium, (2) calcitriol alone increased the enzyme more than in combination with curcumin, (3) these findings demonstrate that curcumin can induce early osteogenic differentiation of DPSCs like calcitriol as a potent stimulant of osteogenesis, and (4) more *in vitro* and *in vivo* studies are required to confirm their influence on osteo/odontogenic differentiation.

## Data Availability

The raw/processed data required to reproduce these results are available upon request.
